# Impact of Amendments on the Physical Properties of Soil under Tropical Long-Term No Till Conditions

**DOI:** 10.1371/journal.pone.0167564

**Published:** 2016-12-13

**Authors:** Antonio C. A. Carmeis Filho, Carlos A. C. Crusciol, Tiara M. Guimarães, Juliano C. Calonego, Sacha J. Mooney

**Affiliations:** 1 Department of Crop Science, College of Agricultural Sciences, São Paulo State University (UNESP), Botucatu, State of São Paulo, Brazil; 2 Division of Agricultural & Environmental Sciences, The University of Nottingham, Sutton Bonington, Nottingham, United Kingdom; RMIT University, AUSTRALIA

## Abstract

Tropical regions have been considered the world’s primary agricultural frontier; however, some physico-chemical deficiencies, such as low soil organic matter content, poor soil structure, high erodibility, soil acidity, and aluminum toxicity, have affected their productive capacity. Lime and gypsum are commonly used to improve soil chemical fertility, but no information exists about the long-term effects of these products on the physical attributes and C protection mechanisms of highly weathered Oxisols. A field trial was conducted in a sandy clay loam (kaolinitic, thermic Typic Haplorthox) under a no-tillage system for 12 years. The trial consisted of four treatments: a control with no soil amendment application, the application of 2.1 Mg ha^-1^ phosphogypsum, the application of 2.0 Mg ha^-1^ lime, and the application of lime + phosphogypsum (2.0 + 2.1 Mg ha^-1^, respectively). Since the experiment was established in 2002, the rates have been applied three times (2002, 2004, and 2010). Surface liming effectively increased water-stable aggregates > 2.0 mm at a depth of up to 0.2 m; however, the association with phosphogypsum was considered a good strategy to improve the macroaggregate stability in subsoil layers (0.20 to 0.40 m). Consequently, both soil amendments applied together increased the mean weight diameter (MWD) and geometric mean diameter (GMD) in all soil layers, with increases of up to 118 and 89%, respectively, according to the soil layer. The formation and stabilization of larger aggregates contributed to a higher accumulation of total organic carbon (TOC) on these structures. In addition to TOC, the MWD and aggregate stability index were positively correlated with Ca^2+^ and Mg^2+^ levels and base saturation. Consequently, the increase observed in the aggregate size class resulted in a better organization of soil particles, increasing the macroporosity and reducing the soil bulk density and penetration resistance. Therefore, adequate soil chemical management plays a fundamental role in improving the soil’s physical attributes in tropical areas under conservative management and highly affected by compaction caused by intensive farming.

## Introduction

The loss of basic cations due to leaching and crop removal associated with the application of high rates of ammoniacal and nitric fertilizers is considered the primary factor leading to soil acidification [1,2], which affects crop production in several agricultural soils worldwide, such as Ultisols and Oxisols [3]. Typically, the problems resulting from soil acidity are linked to poor chemical fertility, such as aluminum (Al^3+^) toxicity and macronutrient deficiency; however, inadequate soil physical properties may also affect the potential productivity of these soils [1].

Due to their mineral properties, such as a low density of electrical charges on clay minerals, low organic matter content, and low cation exchange capacity, Ultisols and Oxisols are susceptible to surface crusting and subsoil compaction, reducing their macroporosity and hydraulic conductivity, which increases the risk of erosion and land degradation [4]. Therefore, management interventions play an important role in enhancing the physical quality of these soils.

Economically, the application of limestone is the most feasible strategy for alleviating soil acidity and for correcting chemical deficiencies [3]. In addition to its effect on pH, lime is a good source of Ca and Mg and can effectively increase fertilizer efficiency. However, due to the chemical characteristics of limestone, such as low solubility and dissolution rates, the method of product incorporation has been recommended to improve the reaction time in soil subsurface layers [5]. According to conservation principles, soil disturbances severely damage the soil structure, accelerating erosion and land degradation processes [6]; therefore, in areas under a no-tillage (NT) system, liming is usually performed superficially [1,7]. Studies conducted by Caires et al. [8], Castro and Crusciol [9] and Caires et al. [2] showed the feasibility of surface liming in improving soil chemical attributes and grain yields; however, according to these authors, the magnitude of the effect varies according to soil texture, lime rates, reaction time, crop rotation, water regime, and the combined use with more soluble materials, such as phosphogypsum. In a tropical area under a no-tillage system and with a minimal disturbance of soil, Crusciol et al. [10] suggested the technique of combining both soil amendments as a good strategy to neutralize Al^3+^ toxicity and to increase the number of basic cations available in subsoil layers in a shorter period compared to an application of lime alone.

Despite studies that reveal the benefits of lime and phosphogypsum on soil chemical properties and crop grain production [11,12], there have been conflicting results regarding the effects of soil amendments on the physical attributes of highly weathered soils. In a short-term study, Roth and Pavan [7] reported that lime incorporation increased clay dispersion and lowered infiltration rates of an Oxisol soil, and these effects were attributed to lower Al^3+^ and H^+^ activities in the soil solution, promoting the compression of the diffuse double layer and the flocculation of particles. Due to the effect of reducing free Al^3+^, the authors also observed clay dispersion effects in treatments with phosphogypsum. Haynes and Naidu [13] suggested that higher Ca^2+^ levels in the soil solution might cause compression of the double layer, consequently promoting particle flocculation; therefore, the application of materials as Ca sources may favor aggregation mechanisms, which are important for improving the soil structure.

In Oxisols and Ultisols, the soil organic matter content has a profound effect on the soil’s physical attributes because physico-chemical reactions with inorganic compounds play a fundamental role in aggregate formation and soil structure [14]. Roots, fungal hyphae, humic substances, soil organisms, and polysaccharides are important binding agents involved in aggregate formation [15,16], and because these fractions can be influenced by agricultural management [13], the long-term benefits of amendment practices may improve the structure of the entire soil profile, including deeper soil layers.

Based on this information, the following hypotheses were proposed: a) surface liming can increase the water stability of aggregates because of the effect on the cation exchange capacity and Ca availability; b) soil acidity alleviation can influence several mechanisms acting on the formation and stability of aggregates; c) the addition of phosphogypsum in lime-amended soil can favor root growth, which is an important source of biocompounds involved in soil aggregation and porous structure formation; and d) the application of soil amendments can favor C protection mechanisms. There is no information regarding the effect of both soil amendments on improving the physical fertility of the tropical soil profile under a long-term no-tillage system. This study aimed to evaluate the long-term effect of the superficial application of lime and phosphogypsum on the soil’s physical attributes and on the protection mechanisms of total organic carbon (TOC) in a tropical Oxisol under an NT system in a dry winter region.

## Materials and Methods

### Site description

The experiments were conducted in Botucatu, SP, Brazil (48° 23′ W, 22° 51′ S and 765 m) in an area under a no-till system over a 12-year period. The soil was classified as a sandy clay loam (kaolinitic, thermic Typic Haplorthox) [17]. According to Köeppen's classification, the climate is Cwa, with a dry winter and a hot, wet summer. The rainfall values as well as the mean maximum and minimum temperatures recorded over a long period (50 years) are shown in Table 1. Prior to establishing the experiment in 2002, the chemical and physical properties were determined (0–0.2 m), according to the methodology proposed by van Raij et al. [18] and Kiehl [19], respectively. The following results were obtained: organic matter, 21 g dm^-3^; pH (1:2.5 soil/CaCl_2_ suspension 0.01 mol L^-1^), 4.2; P (resin), 9.2 mg dm^-3^; exchangeable K, Ca, and Mg 1.2, 14, and 5 mmol_c_ dm^-3^, respectively; total acidity at pH 7.0 (H + Al) 37 mmol_c_ dm^-3^, cation exchange capacity (CEC) 57 mmol_c_ dm^-3^; base saturation 35%; sand, silt, and clay contents of 54, 11, and 35%, respectively. In the subsoil (0.20–0.40 m), the clay content was 36%.

**Table 1:** Rainfall and maximum and minimum temperatures in Botucatu, São Paulo, Brazil, over a long period.

Climate characteristics	Month											
	Jan.	Feb.	Mar	Apr.	May	Jun.	Jul.	Aug.	Sep.	Oct.	Nov.	Dec.
	Long-term (50-yr) avg.											
Monthly rain, mm	224.0	203.2	140.9	66.5	75.8	55.9	37.7	38.9	71.3	126.5	133.3	184.6
Mean max. temp., °C	28.1	28.0	28.0	27.0	24.0	23.0	23.0	25.0	26.2	26.7	27.2	27.2
Mean min. temp., °C	17.1	17.4	19.0	17.0	15.0	13.0	13.0	14.0	12.4	14.2	15.1	16.4

### Experimental design and treatment establishment

A complete randomized block design was used with four replications. Each plot covered an area of 46.8 m^2^ (5.2 m x 9.0 m). The plots were composed of four treatments: (i) a control (no lime or phosphogypsum), (ii) phosphogypsum (2.1 Mg ha^-1^), (iii) lime (2.0 Mg ha^-1^) and (iv) lime + phosphogypsum (2.0 Mg ha^-1^ + 2.1 Mg ha^-1^, respectively). The dolomitic limestone (Embracal^®^, Saltinho, São Paulo, Brazil) was composed of 23.3% CaO and 17.5% MgO, and the rate (R) was calculated using Eq (1) to increase the base saturation in the topsoil (0–0.20 m) to 70%, as described by Cantarella et al. [20]:

$$R(Mg.ha^{-1})=\frac{(BS_{2}-BS_{1}).CEC}{(ECCE/100)}$$ (1)

where ECCE is the effective calcium carbonate equivalent of the dolomite and BS_2_ is the estimated base saturation (70%). BS_1_ is the base saturation determined by the soil chemical analysis and calculated using Eq (2):

$$BS_{1}(\%)=\frac{(Ca_{ex}+Mg_{ex}+K_{ex}).100}{CEC}$$ (2)

where Ca_ex_, Mg_ex_, and K_ex_ are the levels of basic exchangeable cations in the soil. CEC is the total cation exchange capacity, calculated according to Eq (3):

$$CEC(mmol_{c}.dm^{-3})=Ca_{ex}+Mg_{ex}+K_{ex}+(H+Al)$$ (3)

The phosphogypsum (Embracal^®^, Saltinho, São Paulo, Brazil) was composed of 20% Ca, 16% S and residual P and F (0.1%). Minor heavy metal contents were detected, including Cd, Ni, Pb, Cr, and Hg at levels of 3.1, 53.6, 12.6, 990, and <0.1 mg kg^-1^, respectively, according to the US Environmental Protection Agency definition of trace element pollution levels in by-product materials. The phosphogypsum rate (PR) was calculated using Eq (4), according to the method proposed by van Raij et al. [21]:

$$PR(Mg.ha^{-1})=6.CL$$ (4)

where CL is the clay content (g kg^–1^) in the soil layer at a 0.20- to 0.40-m depth.

During the study period, the treatments were applied three times, and different species were cropped in season and off season from 2002 to 2015. Details of the crop sequences and fertilizer management are shown in Table 2.

**Table 2:** Crops grown during the study period and the treatment application scheme during the experimental period (from 2002 to 2015).

Season	Crops		Treatment application
	Summer crop	Autumn-winter-spring crop	
**2002/2003**	***Oriza sativa* (cv. Caiapó)** BF: 300 kg ha^–1^ of NPK 08–28–16 + 4.5% S + 0.5% Zn. TF: 50 kg of N ha^–1^	***Avena strigosa* (cv. Comum)** BF: 200 kg ha^–1^ of NPK 10–20–10 + 4.5% S	Lime: 2,700 kg ha^-1^ (71% ECCE)† Gypsum: 2,100 kg ha^-1^
**2003/2004**	***Phaseolus vulgaris* (cv. Pérola)** BF: 300 kg ha^–1^ of NPK 08–28–16 + 4.5% S + 0.5% Zn. TF: 110 kg of N ha^–1^	***Avena strigosa* (cv. Comum)** BF: 200 kg ha^–1^ of NPK 04–20–20 + 7% S	
**2004/2005**	***Arachis hypogaea* (cv. Runner IAC 886)** BF: 24 kg ha^–1^ of N + 84 kg ha^–1^ P_2_O_5_ + 48 kg ha^–1^ of K_2_O + 10% S + 0.5% Zn.	***Avena sativa* (cv. IAC 7)** BF: 300 kg ha^–1^ of NPK 08–28–16 + 4.5% S + 0.5% Zn. TF: 110 kg of N ha^–1^	Lime: 2,000 kg ha^-1^ (71% ECCE) Gypsum: 2,100 kg ha^-1^
**2005/2006**	***Arachis hypogaea* (cv. Runner IAC 886)** BF: 24 kg ha^–1^ of N + 84 kg ha^–1^ P_2_O_5_ + 48 kg ha^–1^ of K_2_O + 10% S + 0.5% Zn.	***Avena sativa* (cv. IAC 7)** BF: 8 kg ha^–1^ of N + 40 kg ha^–1^ P_2_O_5_ + 20 kg ha^–1^ of K_2_O + 7% S	
**2006/2007**	***Zea mays* (cv. 2B570)** BF: 24 kg ha^–1^ of N + 84 kg ha^–1^ P_2_O_5_ + 48 kg ha^–1^ of K_2_O + 10% S + 0.5% Zn. TF: 90 kg of N ha^–1^	***Urochloa brizantha* (cv. Marandu)** The forage seeds were simultaneously sown with corn.	
**2007/2008**	***Zea mays* (cv. 2B570)** BF: 24 kg ha^–1^ of N + 84 kg ha^–1^ P_2_O_5_ + 48 kg ha^–1^ of K_2_O + 10% S + 0.5% Zn. TF: 90 kg of N ha^–1^	***Urochloa brizantha* (cv. Marandu)** The forage seeds were simultaneously sown with corn.	
**2008/2009**	***Glycine max* (cv. MGBR-46)** BF: 250 kg ha^–1^ of NPK 04–20–20 + 4.5% S + 0.5% Zn.	***Avena strigosa* (cv. Comum)** No fertilizer was applied	
**2009/2010**	***Glycine max* (cv. CD216)** BF: 250 kg ha^–1^ of NPK 04–20–20 + 4.5% S + 0.5% Zn.	***Sorghum vulgare* (cv. AG1020)** No fertilizer was applied	
**2010/2011**	***Zea mays* (cv. 2B433)** BF: 350 kg ha^–1^ of NPK 08–28–16. TF: 150 kg of N ha^–1^	***Crambe abyssinica* (cv. FMS Brilhante)** BF: 150 kg ha^–1^ of NPK 08–28–16	Lime: 2,000 kg ha^-1^ (88% ECCE) Gypsum: 2,100 kg ha^-1^
**2011/2012**	***Zea mays* (cv. 2B433)** BF: 350 kg ha^–1^ of NPK 08–28–16. TF: 150 kg of N ha^–1^	***Crambe abyssinica* (cv. FMS Brilhante)** BF: 150 kg ha^–1^ of NPK 08–28–16	
**2012/2013**	***Pennisetum glaucum* (cv. ADR300)** No fertilizer was applied	***Triticum aestivum* (cv. CD116)** BF: 35 kg ha^–1^ of N + 70 kg ha^–1^ P_2_O_5_ + 40 kg ha^–1^ of K_2_O + 11 kg of ha^–1^ S + 12 kg of ha^–1^ Zn.	
**2013/2014**	***Phaseolus vulgaris* (cv. Pérola)** BF: 10 kg ha^–1^ of N + 50 kg ha^–1^ P_2_O_5_ + 50 kg ha^–1^ of K_2_O + 11 kg of ha^–1^ S + 12 kg of ha^–1^ Zn. TF: 100 kg of N ha^–1^	***Triticum aestivum* (cv. CD116)** BF: 35 kg ha^–1^ of N + 70 kg ha^–1^ P_2_O_5_ + 40 kg ha^–1^ of K_2_O + 11 kg of ha^–1^ S + 12 kg of ha^–1^ Zn.	
**2014/2015**	***Phaseolus vulgaris* (cv. Pérola)** BF: 10 kg ha^–1^ of N + 50 kg ha^–1^ P_2_O_5_ + 50 kg ha^–1^ of K_2_O + 11 kg of ha^–1^ S + 12 kg of ha^–1^ Zn. TF: 100 kg of N ha^–1^		

^†^ The reapplications in October of 2004 and 2010 were performed when the standard treatment (calculated rate) reached base saturation ≤ 50%. BF: base fertilization; TF: topdressing fertilization.

Additional details about the accumulated shoot and root dry matter of wheat and common bean cropped in the 2013/2014 and 2014/2015 growing seasons, respectively (Table 2), exchangeable Al and Ca, and soil pH for each soil layer (determined two months after the wheat harvest) as a function of the treatments are shown in Table 3. The soil chemical attributes were measured according to the methodology proposed by van Raij et al. [18].

**Table 3:** Accumulated shoot and root dry matter (wheat and common bean), exchangeable Al and Ca, and soil pH at several depths as a function of the surface application of lime and phosphogypsum in a tropical Oxisol under a no-tillage system.

	Treatments			
	Control	Phosphogypsum	Lime	Lime + Phosphogypsum
	**Shoot dry matter accumulated (wheat + common bean), kg ha**^**-1**^			
Total	2,001.5	2,164.7	4,910.5	5,064.1
Soil layer (m)				
	**Root dry matter accumulated (wheat + common bean), g m**^**-3**^			
0–0.05	139.0	174.2	508.0	533.1
0.05–0.10	107.4	134.4	415.9	473.2
0.10–0.20	62.6	45.1	129.5	125.7
0.20–0.40	12.9	14.0	108.2	112.7
0.40–0.60	2.7	5.6	40.0	28.9
	**Exchangeable Al, mmol**_**c**_ **dm**^**-3**^			
0–0.05	20.0	17.9	3.6	0.0
0.05–0.10	26.7	20.7	8.4	0.8
0.10–0.20	26.5	22.7	13.8	6.4
0.20–0.40	30.2	25.6	21.9	18.6
0.40–0.60	32.7	28.5	27.8	28.1
	**Exchangeable Ca, mmol**_**c**_ **dm**^**-3**^			
0–0.05	4.9	7.0	22.5	36.4
0.05–0.10	2.1	4.6	16.6	27.3
0.10–0.20	1.6	3.2	10.4	17.8
0.20–0.40	1.7	3.0	6.6	9.7
0.40–0.60	2.3	2.8	4.0	6.6
	**Soil pH**			
0–0.05	3.8	3.9	4.6	5.3
0.05–0.10	3.7	3.8	4.5	5.1
0.10–0.20	3.7	3.8	4.2	4.6
0.20–0.40	3.8	3.9	4.1	4.2
0.40–0.60	3.9	3.9	4.1	4.1

### Soil sampling

Twelve years after treatment establishment, in 2014 (two months after the wheat harvest), a trench measuring 0.50 m wide, 0.80 m deep and 0.80 m long was dug in each plot. Soil samples were randomly collected using the trench profile. To determine the aggregate stability and particle density at each soil layer, four clod samples were excavated from the trench wall at depth layers of 0.00–0.05, 0.05–0.10, 0.10–0.20, 0.20–0.40, and 0.40–0.60 m to form a composite sample as described by Castro Filho et al. [22]. On the same day, using volumetric rings (height 5.0 cm, internal diameter 4.8 cm), two undisturbed samples were collected at the center of each soil layer to determine the soil bulk density, the total, macro-, and micro-porosity, and the soil penetration resistance (PR) [23].

### Soil physical attributes and C analysis

For the soil’s water-stable aggregate analysis, undisturbed soil monolith samples were air-dried, gently sieved through 8.0-mm sieves and retained on a 4.0-mm sieve. A 25-g aliquot of the retained air-dried aggregate fraction was pre-wetted (using a spray bottle) and was subjected to a wet-sieving procedure for 15 min using a nest of sieves with 4.00, 2.00, 1.00, 0.50, 0.25, and 0.105-mm mesh sizes linked to mechanical equipment adjusted to 31 vertical oscillations min^-1^ [24]. The procedure was performed on six separate classes of water-stable aggregates: >2.0–8.0 mm, >1.0–2.0 mm, >0.5–1.0 mm, >0.25–0.5 mm, >0.105–0.25 mm, and <0.105 mm. The fractions retained on each sieve were transferred to aluminum dishes and were dried in a forced air oven at 45°C for 72 h for later weighing. Based on the weight and initial soil moisture content of each fraction, the values were adjusted to a dry soil weight, which was used to calculate the aggregate distribution (AD%), aggregate stability index (ASI) mean weight diameter (MWD) and geometric mean diameter (GMD), as follows in Eqs (5), (6), (7) and (8), respectively, as described by Kemper and Chepil [24]:

$$AD\%=\frac{\left(100.wi\right)}{\sum wi}$$ (5)

$$ASI\%=\frac{(yi-zi).100}{yi}$$ (6)

$$MWD=\sum_{i=1}^{n}\left(xi.wi\right)$$ (7)

$$GMD=\text{exp}\left(\frac{\sum wi\text{ln}xi}{\sum wi}\right)$$ (8)

where *wi* is the weight of the aggregates of each size class; *xi* is the mean diameter of the size class (mm); *yi* is the weight of the dry soil sample (105°C); and *zi* is the weight of aggregates smaller than 0.25 mm.

To evaluate the amount of TOC within the soil aggregate classes, the fractions were classified into three groups according to the classification proposed by Dube et al. [25]: >2.0–8.0 mm (macroaggregates), >0.25–2.0 mm (mesoaggregates) and >0.105–0.25 mm (microaggregates). The samples of each group were ground in a porcelain mortar and homogenized, and then, the C content was determined using an elemental analyzer.

In the laboratory, the undisturbed samples were saturated by gradually increasing the water level for 48 h. Then, all saturated samples were weighed and subjected to a 0.006-MPa tension on porous plates in Richard’s pressure chamber [26]. Upon reaching stability, the samples were weighed and subjected to a 0.03-MPa tension. Once the samples were at equilibrium, PR analysis was performed using a Marconi electronic penetrometer (model MA933) constructed with a metal vertical probe with a 30° cone and a base area of 0.1256 cm^2^. Penetration into the samples was performed with a constant penetration velocity of 10 mm min^-1^, using a charge cell of 20 kg, to a depth of 40 mm. PR data were acquired with TexturePro CT V1.4 software, and the measurements obtained from the surface of the sample to a 10-mm depth were discarded, as suggested by Tormena et al. [27]. After the PR test, the samples were dried in a forced air oven at 105°C for 48 h and weighed to determine the soil bulk density. The total porosity was the difference between the weighed samples (water-saturated and dried), macroporosity was determined as the water content difference between the water-saturated samples and those subjected to a 0.006-MPa tension, and microporosity was calculated by the difference between the total porosity and macroporosity [28].

The particle density (PD) was obtained using a 20-g aliquot of disturbed samples, which were dried in a forced air oven at 105°C for 24 h. Dried samples were transferred to a 50-ml volumetric flask, and the volume was adjusted with ethyl alcohol (95% v/v) while shaking gently to remove air bubbles. The particle density was calculated using Eq (9), according to the method proposed by Blake and Hartge [29]:

$$PD(kg.dm^{-3})=\frac{x}{\left(50-y\right)}$$ (9)

where *x* is the weight of the dried samples (105°C) and *y* is the ethyl alcohol volume used.

### Statistical analyses

All data were initially tested for normality using the Shapiro-Wilk test from the UNIVARIATE procedure of SAS (version 9.3; SAS Inst. Inc., Cary, NC), and the results indicated that all data were distributed normally (W ≥ 0.80). The data were then analyzed using the PROC MIXED procedure of SAS and the Satterthwaite approximation to determine the degrees of freedom for the tests of fixed effects. The treatments were considered fixed effects. Significant differences between the means were determined using Fisher's protected LSD test. Effects were considered significant at P ≤ 0.05.

## Results

**Fig 1:**
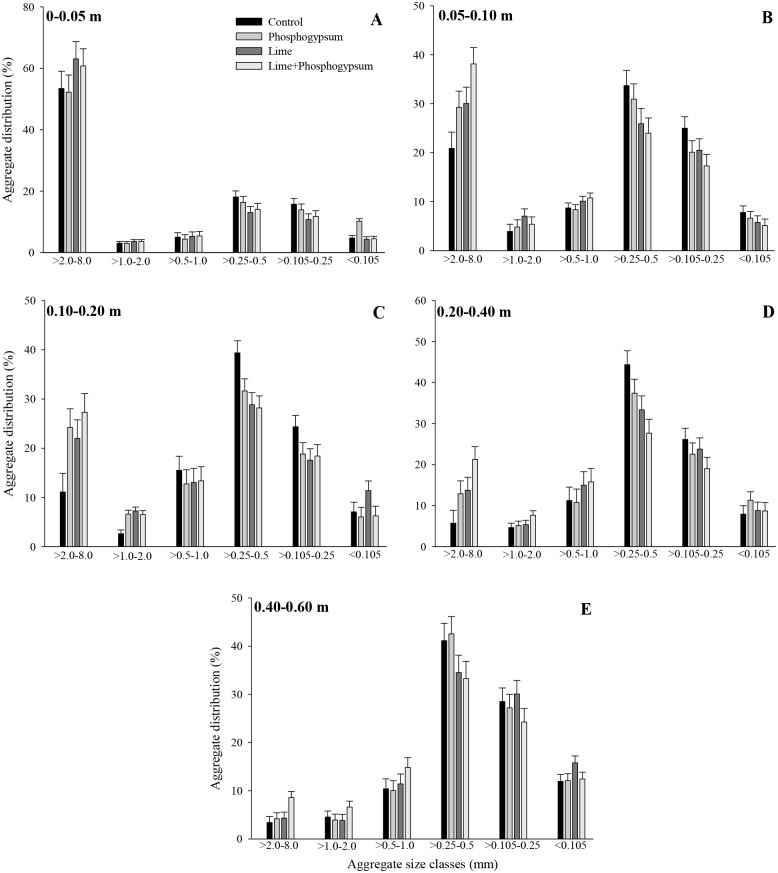
Water-stable aggregate distribution as affected by the surface application of lime and phosphogypsum in different soil layers in a tropical no-tillage system. The vertical bars indicate the least significant difference at p ≤ 0.05.

**Fig 2:**
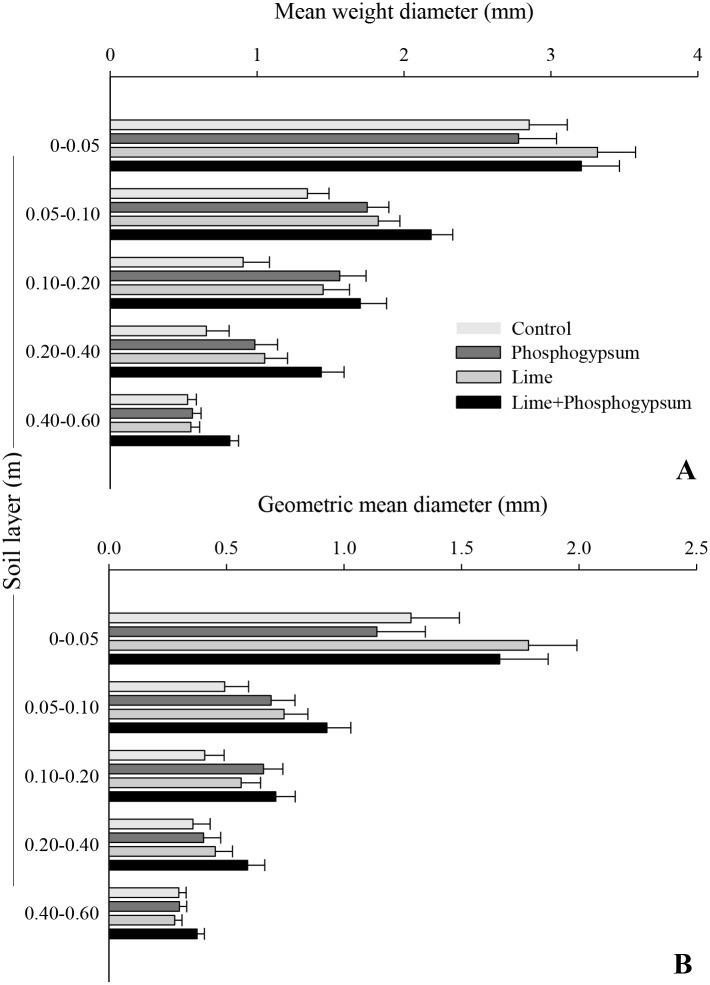
Mean weight diameter and geometric mean diameter of aggregates as affected by the surface application of lime and phosphogypsum in different soil layers in a tropical no-tillage system. The horizontal bars indicate the least significant difference at p ≤ 0.05.

The results showed significant effects (p ≤ 0.05) of soil amendments on the soil physical attributes and on the stabilization of organic carbon via aggregation mechanisms (Tables 4 and 5). The surface application of the combination of lime and phosphogypsum increased the amount of water-stable aggregates >2.0 mm, consequently reducing the classes of >0.25–0.5 and >0.105–0.25 mm in all soil layers (Fig 1, S1 Table). Compared with the control, the effect of association on both soil amendments was more pronounced in the subsurface (below 0.05 m), and the observed increase was 82, 145, 273, and 152% in the 0.05–0.10-, 0.10–0.20-, 0.20–0.40-, and 0.40–0.60-m soil layers, respectively. Liming was the only practice that increased the level of aggregation (classes larger than 1.0 mm), but the product alone had no positive influence on increasing the proportion of macroaggregates in the 0.40–0.60-m layer. At a 0.05- to 0.40-m depth, a reduced effect of the application of phosphogypsum alone was observed, primarily in the aggregate classes of >2–8 mm and >0.105–0.25 mm, which showed increased MWD and GMD (Fig 2, S2 Table).

**Table 4:** ANOVA significance for soil physical attributes and total organic carbon (TOC) content in the water-stable aggregate classes.

Soil layer (m)	F probability		F probability	
	Blocks	Treatments	Blocks	Treatments
	Aggregate-size (>2.0–8.0 mm)		Mean weight diameter	
0–0.05	0.5458	0.0040	0.4979	0.0025
0.05–0.10	0.2906	<0.0001	0.2762	<0.0001
0.10–0.20	0.7876	<0.0001	0.4353	<0.0001
0.20–0.40	0.4396	<0.0001	0.5231	<0.0001
0.40–0.60	0.2538	<0.0001	0.1666	<0.0001
	Aggregate-size (>1.0–2.0 mm)		Geometric mean diameter	
0–0.05	0.4151	0.0524	0.6207	0.0002
0.05–0.10	0.6515	0.0065	0.2232	<0.0001
0.10–0.20	0.8471	<0.0001	0.3541	0.0001
0.20–0.40	0.7006	0.0007	0.1905	0.0002
0.40–0.60	0.9271	0.0021	0.2091	0.0004
	Aggregate-size (>0.5–1.0 mm)		Penetration resistance	
0–0.05	0.8771	0.4191	0.1499	<0.0001
0.05–0.10	0.8540	0.0009	0.9612	0.0246
0.10–0.20	0.9840	0.2724	0.9960	0.0088
0.20–0.40	0.3032	0.0141	0.4761	0.2264
0.40–0.60	0.5779	0.0015	0.6347	<0.0001
	Aggregate-size (>0.25–0.5 mm)		TOC in macroaggregates (>2.0–4.0 mm)	
0–0.05	0.3058	0.0010	0.8736	0.0003
0.05–0.10	0.8010	0.0015	0.5062	0.0002
0.10–0.20	0.5053	<0.0001	0.2856	0.4491
0.20–0.40	0.2961	<0.0001	0.3151	0.0334
0.40–0.60	0.4827	0.0005	0.9912	0.2712
	Aggregate-size (>0.105–0.25 mm)		TOC in mesoaggregates (>0.25–2.0 mm)	
0–0.05	0.5013	0.0009	0.6179	<0.0001
0.05–0.10	0.2795	<0.0001	0.1302	<0.0001
0.10–0.20	0.4863	0.0004	0.6788	<0.0001
0.20–0.40	0.3602	0.0049	0.5174	0.0004
0.40–0.60	0.1449	0.0069	0.3126	0.0005
	Aggregate-size (<0.105 mm)		TOC in microaggregates (>0.105–0.25 mm)	
0–0.05	0.1804	<0.0001	0.1409	0.4698
0.05–0.10	0.7101	0.0075	0.4096	0.0108
0.10–0.20	0.1554	0.0005	0.2187	<0.0001
0.20–0.40	0.7024	0.0216	0.5365	0.0064
0.40–0.60	0.9636	0.0075	0.3877	0.0004

**Table 5:** Aggregate stability index, soil bulk and particle density, total porosity, macroporosity, and microporosity of soil as affected by the surface application of lime and phosphogypsum in different soil layers in a tropical no-tillage system.

Treatments	Soil layers (m)				
	0–0.05	0.05–0.10	0.10–0.20	0.20–040	0.40–0.60
	Aggregate stability index (%)				
Control	79.3 b†	66.3 c	68.3 c	66.5 b	59.3 b
Phosphogypsum	75.7 c	73.5 b	75.4 a	65.8 b	60.4 b
Lime	85.0 a	73.7 b	71.1 b	67.5 b	54.1 c
Lime + Phosphogypsum	83.8 a	77.6 a	75.4 a	72.3 a	63.3 a
**F probability**					
Block	0.6813	0.5679	0.8518	0.5055	0.3745
Treatments	<0.0001	<0.0001	0.0001	0.0041	<0.0001
	Soil bulk density (kg dm^3^)				
Control	1.60 a	1.78 a	1.79 a	1.73 a	1.56 a
Phosphogypsum	1.54 a	1.69 ab	1.77 a	1.61 b	1.56 a
Lime	1.66 a	1.68 ab	1.62 b	1.53 c	1.39 b
Lime + Phosphogypsum	1.56 a	1.62 b	1.56 b	1.48 c	1.36 b
**F probability**					
Block	0.5089	0.5288	0.3377	0.2837	0.5278
Treatments	0.4473	0.1531	0.0059	<0.0001	0.0016
	Particle density (kg dm^3^)				
Control	2.28 a	2.46 a	2.46 a	2.42 a	2.26 a
Phosphogypsum	2.27 a	2.34 ab	2.46 a	2.32 ab	2.24 a
Lime	2.34 a	2.34 ab	2.32 ab	2.23 b	2.09 b
Lime + Phosphogypsum	2.24 a	2.29 b	2.21 b	2.14 b	2.04 b
**F probability**					
Block	0.2660	0.2958	0.3301	0.2782	0.9001
Treatments	0.5936	0.0710	0.0135	0.0437	0.0045
	Total porosity (%)				
Control	42.0 a	37.8 a	37.1 b	39.5 b	45.1 b
Phosphogypsum	47.1 a	38.8 a	39.3 b	43.7 ab	44.6 b
Lime	41.5 a	39.8 a	43.0 a	46.5 a	50.3 a
Lime + Phosphogypsum	44.1 a	41.6 a	42.0 a	45.3 a	50.6 a
**F probability**					
Block	0.9190	0.2948	0.7681	0.8302	0.2173
Treatments	0.4157	0.4718	0.0041	0.0670	0.0122
	Macroporosity (%)				
Control	12.0 b	6.8 b	5.5 b	6.0 c	10.3 b
Phosphogypsum	16.9 a	10.8 a	8.5 a	14.5 a	11.5 b
Lime	9.0 b	7.0 b	8.7 a	10.5 b	14.3 a
Lime + Phosphogypsum	11.8 b	9.8 a	8.5 a	9.0 b	14.8 a
**F probability**					
Block	0.2635	0.1262	0.6010	0.5886	0.9296
Treatments	0.0023	0.0047	0.0029	0.0001	0.0005
	Microporosity (%)				
Control	30.0 a	31.0 a	31.8 a	33.3 ab	34.8 a
Phosphogypsum	30.3 a	28.0 a	31.0 a	29.0 b	32.8 a
Lime	32.5 a	32.8 a	34.5 a	36.5 a	36.0 a
Lime + Phosphogypsum	32.3 a	31.8 a	33.5 a	36.0 a	35.8 a
**F probability**					
Block	0.6779	0.9699	0.4261	0.8588	0.5265
Treatments	0.7041	0.2948	0.2272	0.0433	0.5257

^†^ Values followed by the same letter within a column are not significantly different at p ≤ 0.05 according to the LSD test.

The soil amendment combination (lime + phosphogypsum) was considered a highly effective technique to improve the level of soil aggregation, providing the highest MWD and GMD values in all soil layers (S2 Table), with a high correlation observed between MWD and soil chemical parameters. Compared with liming alone, the synergistic effect of phosphogypsum applied with lime only was not observed at the soil surface (0- to 0.05-m depth). In addition, the combination of both soil amendments contributed to the increase in MWD and GMD in deeper soil layers.

**Fig 3:**
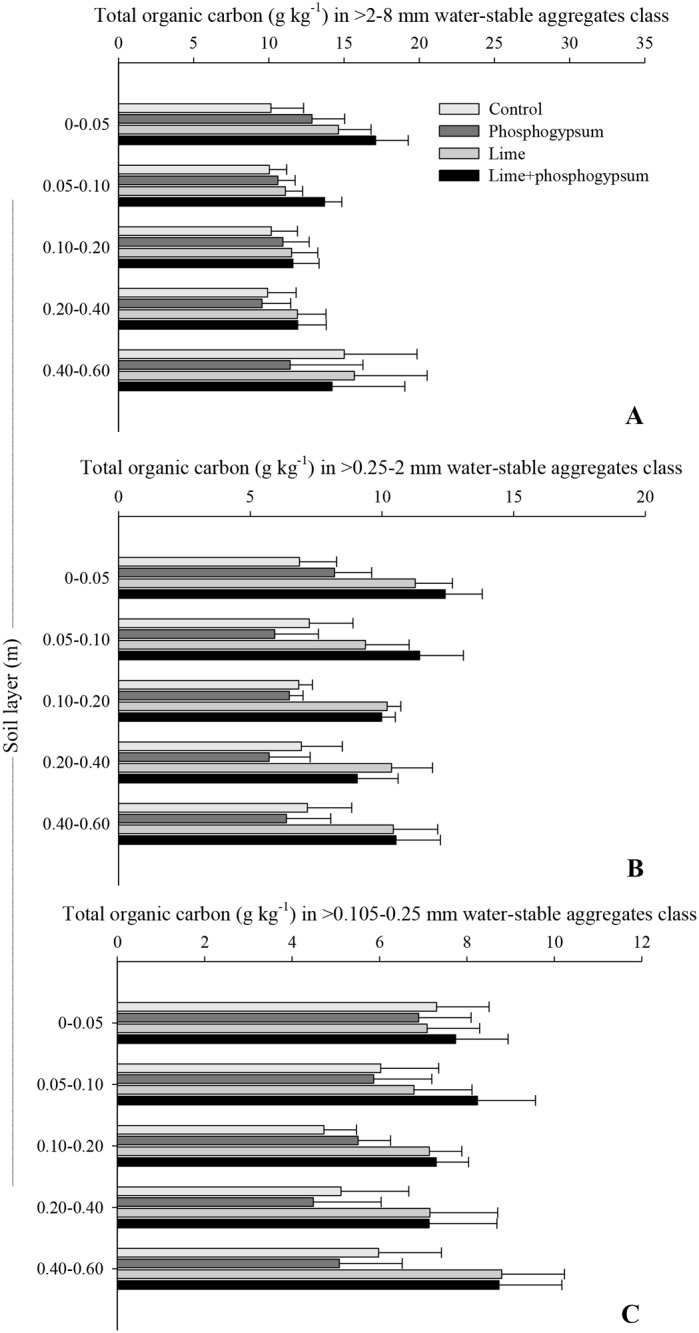
Total organic carbon content in the water-stable aggregate classes as affected by the surface application of lime and phosphogypsum in different soil layers in a tropical no-tillage system. The horizontal bars indicate the least significant difference at p ≤ 0.05.

The combined application of lime and phosphogypsum effectively increased the organic C content in different classes of aggregates (>2.0–8.0, >0.25–2.0, and >0.105–0.25 mm) (Fig 3, S3 Table). In soil surface layers (0–0.05 and 0.05–0.10 m), the amendment materials resulted in the highest accumulation of organic C on macro- and meso-aggregates, i.e., 68 and 80% from 0 to 0.05 m and 76 and 58% from 0.05 to 0.10 m, respectively, compared with the control treatment. A greater TOC content in the microaggregates was also observed; however, only the combination of both soil amendments was considered effective for increasing the TOC content in this class of aggregates in all soil layers because surface liming alone did not increase the TOC in microaggregates at a depth of up to 0.1 m. Despite the positive effect of phosphogypsum on the protection mechanisms and the promotion of organic C accumulation in an Oxisol, the benefits were only effective when phosphogypsum was applied in combination with lime.

The ASI was positively affected by a combination of lime and phosphogypsum, with gains of 6%, 17%, 10%, 8%, and 7% in the aggregate stability in the soil layers of 0–0.05, 0.05–0.10, 0.10–0.20, 0.20–0.40, and 0.40–0.60-m, respectively (Table 5, S4 Table). This finding may result from the direct effect of these materials on increasing the pH and the Ca exchange levels because there was a high correlation between these parameters and the aggregate stability index (S4 Table). The surface application of lime increased the aggregate stability at a depth of up to 0.40 m, but in the deeper layer (from 0.40 to 0.60 m), this practice increased the proportion of unstable aggregates compared with the control treatment. The surface application of lime, alone or combined with phosphogypsum, was considered an efficient strategy to increase macroporosity (pores with a diameter ≥30 μm) in the soil profile, resulting in an increase in total porosity to a depth of 0.10 m, reducing soil bulk density by up to 16% in subsurface soil layers. Microporosity (pores with a diameter <30 μm) was not affected by the addition of both amendments in a combination; however, in a no-till system, management practices that positively influence the macropore distribution are considered fundamental because these pores directly affect root growth, primarily in regions with dry seasons. Some studies consider a critical degree of compaction when the total macroporosity is less than 10% [30,31].

The lower distribution of macropores in lime-amended soil compared with the application of phosphogypsum alone from a 0- to 0.10-m depth was not associated with penetration resistance because the results obtained showed that only surface liming reduced the ratio of penetration resistance by up to 39% in the uppermost soil layers (S4 Table), which may be related to the formation of biopores via biological activity.

**Fig 4:**
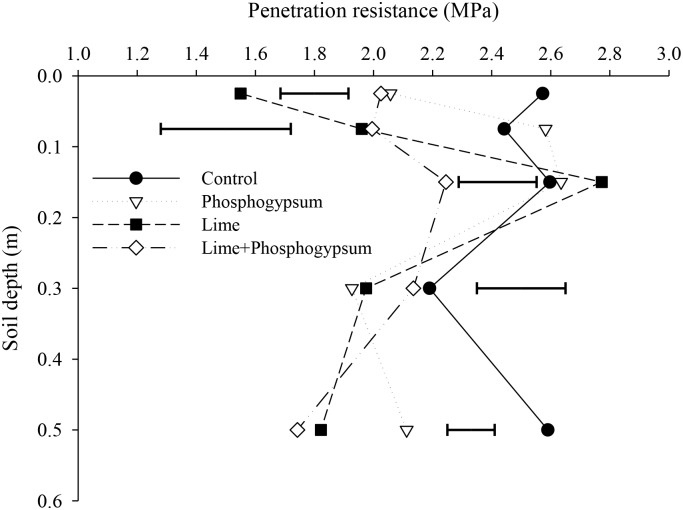
Penetration resistance as affected by the surface application of lime and phosphogypsum in different soil layers in a tropical no-tillage system. The horizontal bars indicate the least significant difference at p ≤ 0.05.

In all soil layers, except at the 0.20–0.40-m depth, lower penetration resistance rates were obtained by applying a combination of lime and phosphogypsum (Fig 4, S5 Table). The application of lime alone had a substantial effect on the uppermost soil layer because this treatment provided the lowest penetration resistance value (1.55 MPa) for a 0- to 0.05-m depth. Both soil amendments, alone or combined, had different effects according to the soil depth but were useful for ameliorating the Oxisol soil structure cultivated under NT systems.

## Discussion

The indirect effect of soil amendments on increasing the input of above- and under-ground residues has been considered to play a fundamental role in improving soil physical attributes (Table 3) [13,32,33]. In a long-term study conducted using a kaolinitic, thermic Typic Hapludox soil, Briedis et al. [33] reported a greater influence of surface liming on the soil structural organization due to the high input of organic C by crop residues and the microbial activity with soil acidity alleviation; however, there is no information available about this influence under tropical conditions. As mentioned, the input of crop residues plays a fundamental role in increasing the formation and stability of larger aggregates, primarily those with a diameter >1 mm [34]; however, in tropical regions, the high temperature and humidity favor the decomposition of organic residues, affecting organic C storage.

Several organic and inorganic compounds increase water-stable aggregation, and according to the aggregate hierarchy concept, each stage of formation is associated with different binding agents [15]. The chemical-physical interaction between some organic molecules and polyvalent cations and mineral particles was proposed by Edwards and Bremner [35] to describe microaggregation (<0.25 mm). Sequentially, due to the action of soil organisms, roots, litter fragments, and non-humic substances (such as carbohydrates), larger aggregates are formed and stabilized [14,36]. Consequently, management practices that enhance SOM input are considered highly effective for the improvement of the soil structure.

Several researchers considered that the high input of labile organic matter (shoot and root fragments) is an important agent for the formation and stability of larger aggregates [13,33,36,37]. Because this organic fraction is highly sensitive to soil acidity and management practices, amendment procedures that improve soil chemical properties without the disruption of soil structure are desirable. In a clay Typic Rhodudalf soil, Castro et al. [9] reported that surface liming could increase labile organic matter in the soil surface layer six years after the treatment establishment; however, the authors did not observe positive results in subsurface layers due to the low effect of lime on acidity alleviation [5]. Roots are considered the most important source of labile organic matter for subsoil layers; thus, practices that increase root growth into subsoil layers play a fundamental role in the formation and stability of larger aggregates in the subsoil. In addition, the root structures are considered a more effective approach to improve the soil structure than above-ground residues [33].

Our long-term results showed the benefits of soil amendments on the stability of the largest aggregate class (>2.0–8.0 mm) (Fig 1), and this effect may be explained by the effect on root growth at deeper soil layers (Table 3). Six et al. [14] reported a strong effect of root development on the proportion of macroaggregates and described the effect on the soil water regime, root exudation, and root biomass as the result of important mechanisms involved in the stability of macroaggregates. Evaluating the effect of root-released substances on aggregate properties over 30 days of incubation, Traoré et al. [38] reported that the proportion of stable aggregates increased 3.8-fold due to the root mucilage effect. Changes in aggregate properties by water regime and microbial community dynamics were reported by Denef et al. [39]. In a soil with 30% of dry weight composed of macroaggregates, these authors reported a reduction in the amount of large aggregates to 21% after the first cycle of drying and wetting, but after two cycles, the macroaggregates became more resistant. In addition, these authors observed a high linear correlation between fungal biomass and the percentage of large aggregates (r^2^ = 0.93), suggesting that the amount of fresh residues via the roots is fundamental to enhance fungal growth, consequently enhancing the structural stability.

In conclusion, due to the effect of soil chemical deficiencies on root growth, the structural stability of the aggregates can be affected. Several studies described Al^3+^ toxicity as the major root growth-limiting factor [12,40]; thus, the modifications caused by lime and phosphogypsum application (alone or combined) on exchangeable Al^3+^ levels may result in differences in the root architecture, thus, in the amounts of stabilization agents. Based on the regression models, Caires et al. [41] observed a highly negative correlation between wheat root length density and exchangeable Al^3+^ levels. At topsoil layers (0–0.20 m), the authors reported a decrease of approximately 6.0% in root length density for each increase of 1 mmol_c_ dm^-3^ in exchangeable Al^3+^. This result can explain the benefits of the lime + phosphogypsum application, showing the highest aggregate stability in the largest aggregate class in the 0.05–0.10-, 0.20–0.40-, and 0.40–0.60-m layers. In a sodic soil with a low infiltration rate, Valzano et al. [42] also reported that the combination of lime and gypsum provided a higher accumulation of SOM, which reduced the degree of dispersion of mineral particles, increasing the proportion of more stable, larger aggregates.

In addition to the indirect effect of the lime + phosphogypsum treatment on enhancing the proportion and stability of macroaggregates (Fig 1), the chemical effects of the different treatments (Table 3) can also induce divergent changes in the flocculation mechanisms. According to Roth and Pavan [7], in soils with variable charges, the addition of lime alone increases the pH, favoring repulsive forces between particles and leading to a dispersion phenomenon; however, because Ca acts as a binding agent, practices that increase Ca levels in the soil enhance the formation and stability of microaggregates, which is essential for large aggregate arrangements. Therefore, the phosphogypsum effect in lime-amended soil can also explain the greater stability of large aggregates, primarily in deeper soil layers (0.20–0.40 and 0.40–0.60 m) in the size classes from >1.0 to 2.0 mm and from >2.0 to 8.0 mm. Baldock et al. [43] reported that dispersive mechanisms in a red-brown soil (Rhodoxeralf) were significantly reduced by the addition of lime and gypsum, but a better effect was observed when higher amounts of Ca^2+^ were added. Corrêa et al. [44] found a significant correlation between Ca and soil aggregation, which was positive for aggregate classes of 4–2 mm and negative for the 0.5–0.25 mm class. Although microaggregate stability plays a fundamental role in macroaggregate arrangements, Baldock et al. [43] emphasized that labile organic matter is essential for the formation and stabilization of larger aggregates because in soil without the addition of organic residues, the authors did not report an effect of lime and gypsum addition on macroaggregate stability.

Due to the better level of organization, a reduction in the proportion of smaller aggregates was observed, primarily in the size classes from >0.105 to 0.25 mm and from >0.25 to 0.5 mm, as reported by Muneer and Oades [45]. Therefore, a higher proportion of water-stable aggregates > 1 mm was observed with soil amendments applied in combination, which resulted in an increase in MWD and GMD (S2 Table). Briedis et al. [33] reported a strong linear relationship between MWD and organic C input (MWD = 0.92 C_input_ + 7.1, R^2^ = 0.99), reinforcing the hypothesis that management techniques that contribute to the increase in soil organic matter (via above- and below-ground biomass) are important for improving the soil structure. In addition to the quantity of C, Martins et al. [36] suggested that the quality of the organic residues might contribute to an increase in the proportion of large water-stable aggregates in Oxisols. The researchers reported a close relationship between MWD and the pentose (r^2^ = 0.88, p<0.001) and hexose (r^2^ = 0.77, p<0.01) contents because these substances stimulate the activity of specific decomposers that are associated with macroaggregation. Because the hydrolysable carbohydrate contents are often associated with soil chemical properties, factors such as soil acidity and nutrient deficiency have limited the supply of these organic molecules [46].

Several organic C compounds, such as humic substances, mucilages, and polysaccharides, are considered important binding agents for the formation and stabilization of aggregates, which provide a protective physical barrier against the action of decomposers, preventing the oxidation of SOM. Each aggregate class is involved in the protection of specific fractions [47]. The fact that most of the organic C is present in the >0.105–0.25 mm water-stable aggregate class is most likely related to the amounts of humic substances because these compounds have a strong chemical interaction with the clay mineral fraction and are considered important binding agents for microaggregation. Due to the high stability of humic compounds, no changes in TOC in the 0–0.05-m layer were observed, and the chemical changes induced by the combined application of lime and phosphogypsum were not enough to induce changes in the SOM dynamics in this layer (Table 3). However, in deeper layers, the soil amendments applied in a combination may influence the metabolic pathway of humic substances, possibly contributing along with the addition of organic residues (via above- and below-ground biomass), mainly those rich in lignin, considered a precursor of these compounds [48]. Roots have been reported to be rich in lignin compared with the aboveground residues [49], and Caires et al. [41] reported the benefits of surface liming on root growth. Our results suggest that soil amendments can modify the amounts of these substances in the soil due to the addition of organic residues, contributing to an increase in TOC in smaller aggregates. Lehmann et al. [50] observed that organic C in soil microaggregates is more closely related to the high stability of these organic molecules on mineral surface charges than to C physical protection.

As mentioned above, the larger aggregates (from >0.25 to 8.0 mm) are formed by the union of small aggregates (from >0.105 to 0.25 mm), and transitory and temporary organic fractions, such as polysaccharides, microorganism cells, and crop residues (roots and shoots), play an important role in this process [51]. Therefore, the major reason that the combined application of lime + phosphogypsum improves TOC stored in larger aggregates (from >0.25 to 2.0 and >2.0 to 8.0 mm) in the surface (0–0.05 and 0.05–0.10 m) and subsurface layers (0.10–0.20 and 0.20–0.40 m) is the effect of soil amendments on root and shoot growth [13] (Fig 3). However, most of the organic C stored in larger aggregates is attributed to aboveground residues (biomass senescence and compounds exuded) [34], and the Al^3+^ complexation and soil acidity alleviation is fundamental for an increase in the C flow. In medium-textured soils, a positive relationship between the amounts of C physically protected and the C input was reported by Chevallier et al. [52]. Because the indirect effect of the surface application of lime and phosphogypsum is to modify different size fractions of SOM [9,13,31], the amendment practices were considered to enhance C storage in tropical acid soils that are naturally low in organic matter content.

The contribution of soil amendments to the formation and stability of large macroaggregates through different SOM fractions has a positive effect on the soil structure over time, primarily increasing the macroporosity and reducing the soil bulk and particle density in subsurface soil layers (below a 0.10-m depth) (Table 5). Similar results were obtained by Haiti et al. [53] in Alfisol soil of sub-humid tropics. The authors primarily attributed the positive effect on the soil’s physical attributes to changes in the TOC content at a 0–0.30-m soil depth, highlighting a significant (P<0.01) and positive linear relationship with total porosity and a negative linear relationship with soil bulk density. Therefore, due to soil amendments application, soil aggregation increased the volume of macropores, which serve as channels for water to flow through a soil profile. Changes in the pore size distribution and SOM content affected the soil bulk and particle density. According to Rühlmann et al. [54], in addition to the quantity of SOM, the factors influencing the composition (ranging from 1.10 to 1.50 g cm^-3^) and the distribution of different organic components in soils significantly affect the soil particle density. Thus, soil acidity management practices in acidic soils are considered a key factor to increase the SOM pool, translating into benefits for the soil’s physical attributes.

The positive effect of phosphogypsum alone on increasing the macroporosity in the uppermost soil layers (0–0.05- and 0.05–0.10-m depths) may be attributed to the ratio between positive and negative charges. As mentioned above, the liming effect of increasing the CEC may induce dispersive forces, which can increase clay dispersion, sealing the surface macropores [7]. Because phosphogypsum does not change the CEC, these dispersive mechanisms were not observed. However, the primary effect is most likely related to the gypsum effect of increasing the root growth, which can affect the volume of biopores formed by root senescence [11,40]. McCallum et al. [55] reported significant benefits from perennial pasture root growth to increase the soil macroporosity (pores >2 mm); in addition, the authors considered management strategies for root growth as an important tool to ameliorate the porosity in undisturbed soil.

Marsili et al. [56] found a significant correlation between the volume of larger pores and soil penetration resistance in the surface layer (0–0.10 m), whereas our results demonstrated the lowest values of penetration resistance in treatments involving the application of lime without gypsum (Table 6). This effect is most likely related to the higher aggregate stability index in lime-amended soil compared with the index in soil treated with only a surface application of phosphogypsum. At the sampling time, phosphogypsum treatments had a higher macroporosity at the soil surface; however, our results suggested that these structures were formed by aggregates with low stability, which characterizes the macropores as fragile structures that are easily affected by anthropogenic and environmental factors. Thus, amendment practices that alleviate soil acidity and increase the amount of stabilizing substances (such as Ca^2+^ levels and crop residues) are considered effective for improving soil structure stability and reducing soil penetration resistance.

**Table 6:** Pearson correlation coefficients among soil physical attributes (aggregate stability index, soil bulk density, mean weight diameter, and penetration resistance) and soil chemical properties (pH, exchangeable Al^3+^, and Ca^2+^) affected by lime and phosphogypsum application in an Oxisol under a long-term no-tillage experiment.

Chemical parameter†	Soil physical attributes							
	Aggregate stability index		Soil bulk density		MWD		Penetration resistance	
	*r*	*P*	*r*	*P*	*r*	*P*	*R*	*P*
pH	0.8851	<0.0001	-0.5600	0.024	0.8728	<0.0001	-0.7899	<0.0001
Al^3+^	-0.9072	<0.0001	0.5926	0.0162	-0.9140	<0.0001	0.8761	<0.0001
Ca^2+^	0.9194	<0.0001	-0.5898	0.0159	0.9014	<0.0001	-0.8025	<0.0001

^†^MWD: mean weight diameter;

In subsoil layers, the results obtained by the lime + phosphogypsum treatment demonstrated the viability of the combined use of both soil amendments. The additive effect of phosphogypsum can be related to the root growth and to the supply of Ca on the subsurface, considering the importance of binding agents between organo-mineral particles. In a Panoche soil irrigated with saline water, Mitchell et al. [57] reported a positive effect of a surface gypsum application on some soil physical attributes, reducing the soil crust strength by an average of 14% and increasing the soil aggregate stability by an average of 46%. However, there is limited information regarding the effect of the combined surface application of lime and phosphogypsum on the physical attributes of the Oxisol profile.

## Conclusion

The surface application of lime and phosphogypsum in a tropical acid soil induced significant changes to the soil physical structure. Our long-term findings showed that the combination of both soil amendments plays an important role in improving the physical properties of tropical acid soils treated with conservative principles, without soil mechanical mobilization. The combined application of lime and phosphogypsum provided a positive effect on the formation and stabilization of large aggregates (>1.0 mm), inducing the complexation of organic carbon in these structures, which is fundamental for the enhancement of soil structure. A better aggregation level promoted larger MWD and GMD, even in deeper soil layers, which may have resulted in greater macroporosity as well as lower soil bulk density and penetration resistance.

## Supporting Information

S1 Table Water-stable aggregate distribution as affected by surface application of lime and phosphogypsum in different soil layers, in a tropical no-tillage system. (PDF)

S2 Table Mean weight diameter and geometric mean diameter of aggregates as affected by surface application of lime and phosphogypsum in different soil layers, in a tropical no-tillage system. (PDF)

S3 Table Total organic carbon in the water-stable aggregate classes as affected by surface application of lime and phosphogypsum in different soil layers, in a tropical no-tillage system. (PDF)

S4 Table Aggregate stability index, soil bulk and particle density, total porosity, macroporosity, and microporosity of soil as affected by the surface application of lime and phosphogypsum in different soil layers in a tropical no-tillage system. (PDF)

S5 Table Penetration resistance as affected by surface application of lime and phosphogypsum in different soil layers, in a tropical no-tillage system. (PDF)

## References

[1] Fageria NK, Nascente AS. Management of soil acidity of South American soils for sustainable crop production. Adv Agron. 2014;128: 221–275.

[2] Caires EF, Haliski A, Bini AR, Scharr DA. Surface liming and nitrogen fertilization for crop grain production under no-till management in Brazil. Europ J Agron. 2015;66: 41–53.

[3] Fageria NK, Baligar VC. Ameliorating soil acidity of tropical oxisols by liming for sustainable crop production. Adv Agron. 2008;99: 345–399.

[4] Lal R. Physical management of soils of the tropics: priorities for the 21st century. Soil Sci. 2000;165: 191–207.

[5] Alcarde JA, Rodella AA. Quality and legislations of fertilizer and acidity correction sources In: Curi N, Marques JJ, Guilherme LRG, Lima JM, Lopes AS, Álvares VH, editors. Topics in soil science (in Portuguese). Viçosa, Brazil: Brazilian Soil Science Society; 2003 pp. 291–334

[6] Leys A, Govers G, Gillijns K, Berckmoes E, Takken I. Scale effects on runoff and erosion losses from arable land under conservation and conventional tillage: the role of residue cover. J Hydrol. 2010;390: 143–154.

[7] Roth CH, Pavan MA. Effects of lime and gypsum on clay dispersion and infiltration in samples of a Brazilian Oxisol. Geoderma. 1991;48: 351–361.

[8] Caires EF, Alleoni LRF, Cambri MA, Barth G. Surface application of lime for crop grain production under a no-till system. Agron J. 2005;97: 791–798.

[9] Castro GSA, Crusciol CAC. Effects of superficial liming and silicate application on soil fertility and crop yield under rotation. Geoderma. 2013;195–196: 234–242.

[10] Crusciol CAC, Artigiani ACCA, Arf O, Carmeis Filho ACA, Soratto RP, Nascente AS, et al Soil fertility, plant nutrition, and grain yield of upland rice affected by surface application of lime, silicate, and phosphogypsum in a tropical no-till system. Catena. 2016;137: 87–99.

[11] Farina MPW, Channon P. Acid-subsoil amelioration: ii. Gypsum effects on growth and subsoil chemical properties. Soil Sci Soc Am J. 1988;52: 175–180.

[12] Caires EF, Garbuio FJ, Churka S, Joris HAW. Use of gypsum for crop grain production under a subtropical no-till cropping system. Agron J. 2011;103: 1804–1814.

[13] Haynes RJ, Naidu R. Influence of lime, fertilizer and manure applications on soil organic matter content and soil physical conditions: a review. Nutr Cycl Agro Ecosyst. 1998;51: 123–137.

[14] Six J, Bossuyt H, Degryze S, Denef K. A history of research on the link between (micro)aggregates, soil biota, and soil organic matter dynamics. Soil Tillage Res. 2004;79: 7–31.

[15] Tisdall JM, Oades JM. Organic matter and water-stable aggregates in soils. J Soil Sci. 1982;33: 141–163.

[16] Mbagwu JSC, Piccolo A. Changes in soil aggregate stability induced by amendment with humic substances. Soil Technol. 1989;2: 49–57.

[17] USDA, United States Department of Agriculture. Soil taxonomy: a basic system of soil classification for making and interpreting soil surveys. 2nd ed Washington DC: USDA, NRCS, Agriculture Handbook, 1999 http://www.nrcs.usda.gov/Internet/FSE_DOCUMENTS/nrcs142p2_051232.pdf Accessed 7 July 2015.

[18] van Raij B, Andrade JC, Cantarella H, Quaggio JA. Chemical analysis for fertility evaluation of tropical soils (in Portuguese). Campinas: Instituto Agronômico (IAC); 2001.

[19] Kiehl EJ. Edaphology manual: soil-plant relationship (in Portuguese) Agronômica Ceres. São Paulo, Brazil: Piracicaba; 1979 264 p.

[20] Cantarella H, van Raij B, Quaggio JA. Soil and plant analyses for lime and fertilizer recommendations in Brazil. Commun Soil Sci Plant Anal. 1998;29: 1691–1706.

[21] van Raij B, Cantarella H, Quaggio JA, Furlani AMC. Recommendations for fertilization and liming in the State of São Paulo, Brazil. 2nd ed IAC, Campinas; 1997 285 p. in Portuguese.

[22] Castro Filho C, Lourenço A, Guimarães MF, Fonseca ICB. Aggregate stability under different soil management systems in a red latosol in the state of Parana, Brazil. Soil Tillage Res. 2002;65: 45–51.

[23] Embrapa, Brazilian Agricultural Research Corporation. Manual for methods of soil analysis. 2nd ed Embrapa, Rio de Janeiro; 1997 212 p. in Portuguese

[24] Kemper WD, Chepil WS. Size distribution of aggregates In: Black CA, Evans DD, White JL, Jesminger LE, Clark FE, editors. Methods of soil analysis. Madison: American Society of Agronomy; 1965 pp. 499–510.

[25] Dube F, Zagal E, Stolpe N, Espinosa M. The influence of land-use change on the organic carbon distribution and microbial respiration in a volcanic soil of the Chilean Patagonia. For Ecol Manag. 2009;257: 1695–1704.

[26] Klute A, Klute A. Water retention: laboratory methods In: Klute A, editor. Methods of soil analysis: physical and mineralogical methods. 2nd ed Madison: American Society of Agronomy; 1986 pp. 635–660.

[27] Tormena CA, Silva AP, Libardi P. Caracterização do intervalo hídrico ótimo de um Latossolo Roxo sob plantio direto. R Bras Ciênc Solo. 1998;22: 573–581.

[28] Smith KA, Mullins CE. Soil analysis: physical methods. New York: Marcel Dekker Inc; 1991.

[29] Blake GR, Hartge KH. Bulk density In: Klute A, editor. Methods of soil analysis: physical and mineralogical methods. 2nd ed Madison: American Society of Agronomy; 1986 pp. 363–376.

[30] Pagliai M, Vignozzi N, Pellegrini S. Soil structure and the effect of management practices. Soil Tillage Res. 2004;79: 131–143.

[31] Reichert JM, Suzuki LEAS, Reinert DJ, Horn R, Häkansson I. Reference bulk density and critical degree-of-compactness for no-till crop production in subtropical highly weathered soils. Soil Tillage Res. 2009;102: 242–254.

[32] Garbuio FJ, Jones DL, Alleoni LRF, Murphy DV, Caires EF. Carbon and nitrogen dynamics in an Oxisol as affected by liming and crop residues under no-till. Soil Sci Soc Am J. 2011; 75: 1723–1730.

[33] Briedis C, Sá JCdM, Caires EF, Navarro JdF, Inagaki TM, Boer A, et al Soil organic matter pools and carbon-protection mechanisms in aggregate classes influenced by surface liming in a no-till system. Geoderma. 2012;170: 80–88.

[34] Garcia RA, Li Y, Rosolem CA. Soil organic matter and physical attributes affected by crop rotation under no-till. Soil Sci Soc Am J. 2013;77: 1724–1731.

[35] Edwards AP, Bremner JM. Microaggregates in soils. J Soil Sci. 1967;18: 64–73.

[36] Martins MR, Angers DA, Corá JE. Non-labile plant C contributes to long-lasting macroaggregation of an Oxisol. Soil Biol Biochem. 2013;58: 153–158.

[37] Elliott ET. Aggregate structure and carbon, nitrogen, and phosphorus in native and cultivated soils. Soil Sci Soc Am J. 1986;50: 627–633.

[38] Traoré O, Groleau-Renaud V, Plantureux S, Tubeileh A, Boeuf-Tremblay V. Effect of root mucilage and modelled root exudates on soil structure. Eur J Soil Sci. 2000;51: 575–581.

[39] Denef K, Six J, Bossuyt H, Frey SD, Elliott ET, Merckx R, et al Influence of dry–wet cycles on the interrelationship between aggregate, particulate organic matter, and microbial community dynamics. Soil Biol Biochem. 2001;33: 1599–1611.

[40] Carvalho MCS, van Raij B. Calcium sulphate, phosphogypsum and calcium carbonate in the amelioration of acid subsoils for root growth. Plant Soil. 1997;192: 37–48.

[41] Caires EF, Garbuio FJ, Churka S, Barth G, Corrêa JCL. Effects of soil acidity amelioration by surface liming on no-till corn, soybean, and wheat root growth and yield. Europ J Agron. 2008;28: 57–64.

[42] Valzano FP, Murphy BW, Greene RSB. The long term effects of lime (CaCO3), gypsum (CaSO4.2H2O), and tillage on the physical and chemical properties of a sodic red-brown earth. Aust J Soil Res. 2001;39: 1307–1331.

[43] Baldock D, Beaufoy G, Clark J. The Nature of Farming—Low Intensity Farming Systems in Nine European Countries. London: Institute for Environmental Policy; 1994.

[44] Corrêa JC, Bull LT, Crusciol CAC, Moraes MH. Oxisol physical attributes affected by surface application of flue dust, aqueous lime, sewage sludges and limestone (in Portugusese with English abstract). R Bras Ciênc Solo. 2009;33: 263–272.

[45] Muneer M, Oades J. The role of Ca-organic interactions in soil aggregate stability III. Mechanisms and models. Aust J Soil Res. 1989;27: 411–423.

[46] Manna M, Swarup A, Wanjari R, Mishra B, Shahi D. Long-term fertilization, manure and liming effects on soil organic matter and crop yields. Soil Tillage Res. 2007;94: 397–409.

[47] Bongiovanni MD, Lobartini JC. Particulate organic matter, carbohydrate, humic acid contents in soil macro- and microaggregates as affected by cultivation. Geoderma. 2006;136: 660–665.

[48] Ogner G, Schnitzer M. Chemistry of fulvic acid, a soil humic fraction, and its relation to lignin. Can J Chem. 1971;49: 1053–1063.

[49] Abiven S, Recous S, Reyes V, Oliver R. Mineralisation of C and N from root, stem and leaf residues in soil and role of their biochemical quality. Biol Fertil Soils. 2005;42: 119–128.

[50] Lehmann J, Kinyangi J, Solomon D. Organic matter stabilization in soil microaggregates: implications from spatial heterogeneity of organic carbon contents and carbon forms. Biogeochemistry. 2007;85: 45–57.

[51] Franzluebbers AJ, Arshad MA. Particulate organic carbon content and potential mineralization as affected by tillage and texture. Soil Sci Soc Am J. 1997;61: 1382–1386.

[52] Chevallier T, Blanchart E, Albrecht A, Feller C. The physical protection of soil organic carbon in aggegates: a mechanism of carbon storage in a Vertisol under pasture and market gardening (Martinique, West Indies). Agric Ecosyst Environ. 2004;103: 375–387.

[53] Hati KM, Swarup A, Mishra B, Manna MC, Wanjari RH, Mandal KG, et al Impact of long-term application of fertilizer, manure and lime under intensive cropping on physical properties and organic carbon content of an Alfisol. Geoderma. 2008;148: 173–179.

[54] Rühlmann J, Körschens M, Graefe J. A new approach to calculate the particle density of soils considering properties of the soil organic matter and the mineral matrix. Geoderma. 2006;130: 272–283.

[55] McCallum MH, Kirkegaard JA, Green TW, Cresswell HP, Davies SL, Angus JF, et al. Improved subsoil macroporosity following perennial pastures. Aust J Exp Agric. 2004;44: 299–307.

[56] Marsili A, Servadio P, Pagliai M, Vignozzi N. Changes of some physical properties of a clay soil following passage of rubber- and metal-tracked tractors. Soil Tillage Res. 1998;49: 185–199.

[57] Mitchell JP, Shennan C, Singer MJ, Peters DW, Miller RO, Prichard T, et al. Impacts of gypsum and winter cover crops on soil physical properties and crop productivity when irrigated with saline water. Agric Water Manag. 2000;45: 55–71.

