# Correction: Impact of Amendments on the Physical Properties of Soil under Tropical Long-Term No Till Conditions

**DOI:** 10.1371/journal.pone.0173011

**Published:** 2017-02-22

**Authors:** Antonio C. A. Carmeis Filho, Carlos A. C. Crusciol, Tiara M. Guimarães, Juliano C. Calonego, Sacha J. Mooney

The incorrect reference is given in the first sentence of the second paragraph under the subheading “Soil physical attributes and C analysis” in the Materials and Methods section.

The correct sentence should read: To evaluate the amount of TOC within the soil aggregate classes, the fractions were classified into three groups according to the classification proposed by Assis et al. (2006): >2.0–8.0 mm (macroaggregates), >0.25–2.0 mm (mesoaggregates) and >0.105–0.25 mm (microaggregates).

The reference is: Assis CP, Jucksch I, Mendonça ES, Neves JCL. Carbon and nitrogen in aggregates of an Oxisol submitted to different use and management systems (in Portuguese). Pesq. Agropec. Bras. 2006; 41: 1541–1550

There are errors in the second and third sentences of the third paragraph in the Results section. The correct second sentence is: In soil surface layers (0–0.05 and 0.05–0.10 m), the amendment materials resulted in the highest accumulation of organic C on macro- and meso-aggregates, i.e., 68 and 80% from 0 to 0.05 m and 36 and 58% from 0.05 to 0.10 m, respectively, compared with the control treatment. The correct third sentence is: A greater TOC content in the microaggregates was also observed; however, only the combination of both soil amendments was considered effective for increasing the TOC content in this class of aggregates in all soil layers, except in 0–0.05 m, because surface liming alone did not increase the TOC in microaggregates at a depth of up to 0.1 m.

There is an error in the Acknowledgments section. The correct sentence is: The authors would like to thank the São Paulo Research Foundation (FAPESP, Proc. 2013/18462-0 and 2014/16712-2) for the financial support and the National Council for Scientific and Technological Development (CNPq) for an award for excellence in research to the second and fourth authors.
