# Supplementary material for: Impact of Amendments on the Physical Properties of Soil under Tropical Long-Term No Till Conditions
Source: PLoS One. 2016 Dec 13;11(12):e0167564. doi: 10.1371/journal.pone.0167564 (PMC5154518; doi:10.1371/journal.pone.0167564)
Supplement: S1 Table — (PDF) [file pone.0167564.s001.pdf]

S1. Water-stable aggregate distribution as affected by surface application of lime and phosphogypsum in different soil layers, in a tropical no-tillage system.

Treatment	Rep	Sieve >2.0-8.0 mm					Sieve >1.0-2.0 mm					Sieve >0.5-1.0 mm					Sieve >0.25-0.5 mm					Sieve >0.105-0.25 mm					<0.105 mm				
		0-0.05 m	0.05-0.10 m	0.10-0.20 m	0.20-0.40 m	0.40-0.60 m	0-0.05 m	0.05-0.10 m	0.10-0.20 m	0.20-0.40 m	0.40-0.60 m	0-0.05 m	0.05-0.10 m	0.10-0.20 m	0.20-0.40 m	0.40-0.60 m	0-0.05 m	0.05-0.10 m	0.10-0.20 m	0.20-0.40 m	0.40-0.60 m	0-0.05 m	0.05-0.10 m	0.10-0.20 m	0.20-0.40 m	0.40-0.60 m	0-0.05 m	0.05-0.10 m	0.10-0.20 m	0.20-0.40 m	0.40-0.60 m
Control	1	52.8	20.4	10.6	4.4	3.3	3.0	3.4	2.1	5.5	3.8	6.1	8.6	14.8	11.6	10.8	20.0	35.3	41.0	45.7	42.8	15.7	25.9	25.4	26.1	27.0	4.3	8.7	8.0	6.8	12.3
	2	53.8	21.4	12.1	6.2	4.3	2.8	4.2	3.0	3.8	4.5	4.4	9.1	16.0	10.8	12.0	16.2	36.3	37.9	43.6	40.5	14.4	22.9	23.4	26.3	28.3	4.5	6.4	6.1	9.3	11.4
	3	52.8	19.4	10.6	5.4	3.3	3.0	3.4	3.1	4.5	4.8	5.1	8.5	14.9	11.6	9.8	20.0	31.2	41.0	45.7	42.8	15.7	25.9	23.2	26.1	29.0	4.3	7.3	8.0	8.9	12.3
	4	54.2	22.2	11.0	6.8	2.7	3.2	4.6	2.3	4.8	5.1	4.5	8.6	15.8	11.0	9.1	16.0	32.0	37.5	42.4	38.4	17.1	25.4	25.4	26.2	29.8	5.9	8.7	6.1	6.8	11.9
Gypsum	1	50.5	27.0	22.7	12.0	4.5	3.2	4.0	7.2	5.3	3.7	5.2	9.0	11.7	11.5	8.2	17.4	31.3	31.0	35.4	42.6	16.2	20.9	18.6	20.3	26.6	10.5	7.7	5.8	12.5	12.2
	2	52.7	28.7	25.8	13.8	5.0	2.7	4.6	6.4	5.0	4.2	3.8	8.4	15.2	10.9	10.3	15.6	31.1	32.4	37.3	41.4	12.5	20.8	19.5	22.4	27.0	10.7	6.4	5.9	10.5	11.2
	3	53.7	28.7	22.7	13.8	4.0	2.7	4.6	6.4	5.0	4.2	3.8	8.4	13.2	10.9	10.3	16.6	30.2	32.0	37.3	41.4	12.5	20.8	19.5	25.0	27.0	9.7	6.4	5.9	10.5	11.2
	4	51.9	32.6	25.7	11.8	3.3	3.3	6.0	6.5	5.0	3.7	4.6	7.6	10.9	9.7	11.3	15.8	31.1	31.0	39.6	44.8	14.4	17.9	17.7	22.4	28.1	10.0	5.9	6.5	11.8	13.9
Lime	1	68.5	32.5	22.0	15.6	5.2	3.1	7.1	7.3	5.8	3.5	5.0	11.1	15.3	15.9	10.8	12.4	23.2	28.5	29.1	32.9	10.5	19.3	18.9	19.5	28.5	3.6	54	9.0	8.2	15.8
	2	63.1	30.1	19.4	13.7	4.3	3.6	8.2	7.2	5.3	3.9	5.3	10.7	12.0	15.0	11.4	13.0	25.9	28.8	33.4	34.5	10.7	20.2	17.6	23.8	30.1	4.3	5.7	11.4	8.8	14.7
	3	62.6	29.1	24.0	11.7	3.6	3.4	6.7	6.9	4.8	4.1	4.7	10.3	10.9	13.9	13.3	13.2	28.8	30.0	36.0	36.7	10.7	21.6	16.3	24.5	29.8	4.4	6.1	13.8	10.1	16.0
	4	58.1	28.6	22.6	14.0	4.2	4.4	6.0	7.6	5.4	4.0	6.1	10.8	13.8	15.2	10.2	13.6	25.7	28.0	34.9	33.9	11.0	21.0	17.5	27.4	32.0	4.9	5.7	11.3	8.2	16.7
Lime + Gypsum	1	65.1	40.3	28.2	24.2	9.0	3.2	4.8	6.6	7.1	7.9	4.1	9.1	11.7	17.8	15.2	13.7	25.8	26.4	26.9	30.3	11.5	16.7	20.1	19.4	21.2	4.2	4.2	4.3	7.6	12.4
	2	61.7	39.9	28.5	23.8	8.6	3.8	4.2	5.8	7.7	7.3	5.4	10.0	12.2	13.8	15.7	14.7	20.8	27.5	25.7	35.6	11.5	16.9	16.4	20.1	23.1	3.9	5.4	5.7	7.3	14.7
	3	53.4	33.7	30.9	18.9	7.0	4.0	6.4	6.5	9.0	5.6	6.7	11.1	16.0	20.4	13.8	14.5	27.5	28.8	27.2	36.3	13.0	18.7	16.6	20.0	29.3	4.6	5.1	7.9	9.6	11.9
	4	62.7	38.6	21.6	17.9	9.9	3.4	6.1	7.1	6.8	5.7	5.3	10.0	13.4	11.1	14.6	13.1	21.8	30.1	30.9	30.7	10.9	17.0	20.5	16.7	23.5	5.1	5.7	7.2	10.2	10.7
