# Supplementary material for: Impact of Amendments on the Physical Properties of Soil under Tropical Long-Term No Till Conditions
Source: PLoS One. 2016 Dec 13;11(12):e0167564. doi: 10.1371/journal.pone.0167564 (PMC5154518; doi:10.1371/journal.pone.0167564)
Supplement: S2 Table — (PDF) [file pone.0167564.s002.pdf]

S2. Mean weight diameter and geometric mean diameter of aggregates as affected by surface application of lime and phosphogypsum in different soil layers, in a tropical no-tillage system.

Treatment	Rep	Mean weight diameter					Geometric mean diameter				
		0-0.05 m	0.05-0.10 m	0.10-0.20 m	0.20-0.40 m	0.40-0.60 m	0-0.05 m	0.05-0.10 m	0.10-0.20 m	0.20-0.40 m	0.40-0.60 m
Control	1	2.84	1.32	0.88	0.61	0.52	1.29	0.48	0.39	0.35	0.30
	2	2.85	1.37	0.94	0.66	0.57	1.28	0.49	0.41	0.36	0.30
	3	2.83	1.27	0.89	0.65	0.54	1.29	0.47	0.39	0.34	0.29
	4	2.89	1.41	0.91	0.70	0.48	1.28	0.53	0.44	0.36	0.30
Gypsum	1	2.71	1.64	1.64	0.94	0.58	1.05	0.61	0.68	0.41	0.31
	2	2.79	1.72	1.49	1.03	0.60	1.14	0.69	0.66	0.40	0.30
	3	2.85	1.72	1.49	1.03	0.55	1.22	0.67	0.62	0.43	0.32
	4	2.77	1.92	1.63	0.94	0.51	1.15	0.79	0.67	0.37	0.27
Lime	1	3.57	1.94	1.56	1.13	0.59	2.00	0.83	0.61	0.53	0.30
	2	3.32	1.81	1.44	1.05	0.55	1.78	0.74	0.56	0.45	0.28
	3	3.29	1.78	1.31	0.95	0.52	1.76	0.68	0.50	0.40	0.27
	4	3.09	1.77	1.49	1.08	0.54	1.60	0.73	0.58	0.43	0.27
Lime + Gypsum	1	3.41	2.27	1.87	1.59	0.84	1.81	1.03	0.79	0.63	0.41
	2	3.26	2.25	1.73	1.54	0.84	1.70	0.93	0.77	0.68	0.39
	3	2.86	2.01	1.76	1.38	0.73	1.42	0.80	0.69	0.54	0.33
	4	3.30	2.21	1.45	1.24	0.85	1.72	0.95	0.59	0.51	0.37
