# Supplementary material for: Impact of Amendments on the Physical Properties of Soil under Tropical Long-Term No Till Conditions
Source: PLoS One. 2016 Dec 13;11(12):e0167564. doi: 10.1371/journal.pone.0167564 (PMC5154518; doi:10.1371/journal.pone.0167564)
Supplement: S3 Table — (PDF) [file pone.0167564.s003.pdf]

S3. Total organic carbon in the water-stable aggregate classes as affected by surface application of lime and phosphogypsum in different soil layers, in a tropical no-tillage system.

Treatment	Rep	TOC in >2-8.0 mm water-stable aggregates class					TOC in >0.25-2.0 mm water-stable aggregates class					TOC in >0.105-0.25 mm water-stable aggregates class				
		0-0.05 m	0.05-0.10 m	0.10-0.20 m	0.20-0.40 m	0.40-0.60 m	0-0.05 m	0.05-0.10 m	0.10-0.20 m	0.20-0.40 m	0.40-0.60 m	0-0.05 m	0.05-0.10 m	0.10-0.20 m	0.20-0.40 m	0.40-0.60 m
Control	1	9.7	9.9	10.2	9.7	17.8	6.3	7.7	6.7	6.2	6.9	7.0	6.3	5.0	5.0	6.1
	2	10.1	10.0	8.3	9.9	9.6	6.9	7.0	6.8	6.9	7.2	6.8	6.0	4.7	5.1	6.0
	3	10.5	9.5	11.1	9.5	16.8	6.5	6.2	5.9	6.6	6.3	7.3	4.7	4.2	4.4	5.1
	4	10.2	10.7	11.1	10.5	15.9	7.8	7.0	7.9	8.0	8.3	8.2	7.1	5.0	6.0	6.7
Gypsum	1	15.2	10.6	10.2	9.6	12.2	9.4	7.3	6.5	6.1	6.6	5.4	5.1	6.3	5.3	4.8
	2	12.9	10.6	10.9	9.6	14.7	8.2	5.9	6.5	5.7	6.4	7.7	5.9	5.5	4.5	5.1
	3	11.0	11.0	11.9	8.3	9.5	8.0	5.3	7.3	6.4	6.8	6.9	5.9	4.3	4.6	5.7
	4	12.4	10.2	10.8	10.7	9.3	7.2	6.2	5.7	4.7	5.7	7.6	6.6	5.9	3.6	4.8
Lime	1	15.2	10.4	11.2	11.4	12.1	11.3	10.0	9.1	11.6	10.8	6.0	6.6	7.0	8.4	9.6
	2	13.4	11.2	12.3	13.4	17.9	11.3	8.4	10.2	9.4	9.2	6.1	6.0	7.1	5.5	7.4
	3	14.9	10.8	11.9	10.6	15.9	11.2	9.2	10.5	9.7	11.2	8.2	6.8	7.0	6.9	9.0
	4	15.0	12.0	10.7	12.2	16.9	11.3	9.9	11.0	10.7	10.5	8.1	7.8	7.4	7.9	9.2
Lime + Gypsum	1	15.2	12.7	10.2	10.2	12.7	11.9	10.7	9.9	8.9	10.9	8.4	8.4	6.8	6.1	7.4
	2	17.1	13.7	11.6	10.3	14.2	12.4	11.4	10.3	7.4	8.3	6.3	9.5	7.8	7.1	7.9
	3	17.4	15.2	11.1	13.3	14.5	11.2	11.1	9.8	9.8	12.7	8.1	7.6	7.3	6.9	9.8
	4	18.7	13.2	13.5	13.8	15.4	14.1	12.5	10.0	10.1	10.2	8.1	7.5	7.4	8.4	9.9
