# Supplementary material for: Impact of Amendments on the Physical Properties of Soil under Tropical Long-Term No Till Conditions
Source: PLoS One. 2016 Dec 13;11(12):e0167564. doi: 10.1371/journal.pone.0167564 (PMC5154518; doi:10.1371/journal.pone.0167564)
Supplement: S4 Table — (PDF) [file pone.0167564.s004.pdf]

S4. Aggregate stability index, soil bulk and particle density, total porosity, macroporosity, and microporosity of soil as affected by surface application of lime and phosphogypsum in different soil layers, in a tropical no-tillage system.

| Treatment | Rep | Aggregate stability index | | | | | Soil bulk density | | | | | Particle density | | | | | Total porosity | | | | | Macroporosity | | | | | Microporosity | | | | |
|---|---|---|---|---|---|---|---|---|---|---|---|---|---|---|---|---|---|---|---|---|---|---|---|---|---|---|---|---|---|---|---|
| | | 0-0.05 m | 0.05-0.10 m | 0.10-0.20 m | 0.20-0.40 m | 0.40-0.60 m | 0-0.05 m | 0.05-0.10 m | 0.10-0.20 m | 0.20-0.40 m | 0.40-0.60 m | 0-0.05 m | 0.05-0.10 m | 0.10-0.20 m | 0.20-0.40 m | 0.40-0.60 m | 0-0.05 m | 0.05-0.10 m | 0.10-0.20 m | 0.20-0.40 m | 0.40-0.60 m | 0-0.05 m | 0.05-0.10 m | 0.10-0.20 m | 0.20-0.40 m | 0.40-0.60 m | 0-0.05 m | 0.05-0.10 m | 0.10-0.20 m | 0.20-0.40 m | 0.40-0.60 m |
| Control | 1 | 80.4 | 66.0 | 67.2 | 67.2 | 60.8 | 1.54 | 1.78 | 1.82 | 1.74 | 1.49 | 2.19 | 2.43 | 2.45 | 2.43 | 2.17 | 0.42 | 0.37 | 0.35 | 0.38 | 0.45 | 0.14 | 0.07 | 0.05 | 0.07 | 0.11 | 0.33 | 0.35 | 0.29 | 0.30 | 0.38 |
| | 2 | 79.3 | 66.3 | 68.2 | 66.5 | 59.3 | 1.64 | 1.85 | 1.87 | 1.77 | 1.63 | 2.35 | 2.52 | 2.56 | 2.46 | 2.35 | 0.44 | 0.36 | 0.37 | 0.38 | 0.44 | 0.12 | 0.05 | 0.05 | 0.05 | 0.11 | 0.29 | 0.28 | 0.34 | 0.34 | 0.34 |
| | 3 | 80.2 | 66.0 | 67.5 | 67.2 | 60.0 | 1.60 | 1.78 | 1.79 | 1.73 | 1.56 | 2.28 | 2.46 | 2.46 | 2.42 | 2.26 | 0.42 | 0.38 | 0.37 | 0.40 | 0.45 | 0.14 | 0.07 | 0.05 | 0.06 | 0.10 | 0.30 | 0.32 | 0.32 | 0.33 | 0.35 |
| | 4 | 77.2 | 66.7 | 70.1 | 65.0 | 57.0 | 1.63 | 1.72 | 1.69 | 1.69 | 1.55 | 2.31 | 2.44 | 2.38 | 2.36 | 2.25 | 0.42 | 0.41 | 0.40 | 0.42 | 0.45 | 0.08 | 0.08 | 0.07 | 0.06 | 0.09 | 0.28 | 0.29 | 0.32 | 0.36 | 0.32 |
| Gypsum | 1 | 74.1 | 70.7 | 75.7 | 66.2 | 58.8 | 1.36 | 1.55 | 1.73 | 1.63 | 1.51 | 2.08 | 2.23 | 2.43 | 2.49 | 2.22 | 0.52 | 0.44 | 0.40 | 0.44 | 0.47 | 0.19 | 0.13 | 0.08 | 0.14 | 0.12 | 0.27 | 0.26 | 0.30 | 0.32 | 0.33 |
| | 2 | 75.7 | 73.5 | 75.4 | 67.1 | 60.4 | 1.59 | 1.69 | 1.76 | 1.62 | 1.55 | 2.31 | 2.47 | 2.46 | 2.33 | 2.24 | 0.45 | 0.36 | 0.40 | 0.44 | 0.44 | 0.16 | 0.09 | 0.09 | 0.13 | 0.12 | 0.31 | 0.33 | 0.32 | 0.31 | 0.34 |
| | 3 | 77.6 | 72.8 | 74.7 | 65.8 | 61.0 | 1.54 | 1.81 | 1.77 | 1.61 | 1.56 | 2.27 | 2.34 | 2.46 | 2.32 | 2.24 | 0.47 | 0.38 | 0.39 | 0.44 | 0.44 | 0.17 | 0.09 | 0.08 | 0.14 | 0.11 | 0.30 | 0.25 | 0.31 | 0.29 | 0.33 |
| | 4 | 75.6 | 77.0 | 75.7 | 64.3 | 61.5 | 1.68 | 1.71 | 1.83 | 1.59 | 1.60 | 2.41 | 2.31 | 2.50 | 2.13 | 2.27 | 0.43 | 0.35 | 0.36 | 0.43 | 0.42 | 0.16 | 0.12 | 0.09 | 0.17 | 0.11 | 0.33 | 0.28 | 0.31 | 0.24 | 0.31 |
| Lime | 1 | 86.3 | 76.1 | 72.3 | 65.8 | 56.0 | 1.63 | 1.61 | 1.54 | 1.50 | 1.43 | 2.30 | 2.24 | 2.22 | 2.08 | 2.14 | 0.43 | 0.39 | 0.44 | 0.49 | 0.50 | 0.09 | 0.07 | 0.10 | 0.10 | 0.13 | 0.32 | 0.33 | 0.36 | 0.37 | 0.38 |
| | 2 | 85.0 | 73.5 | 70.8 | 67.4 | 54.1 | 1.69 | 1.71 | 1.66 | 1.55 | 1.49 | 2.38 | 2.38 | 2.35 | 2.37 | 2.16 | 0.38 | 0.39 | 0.42 | 0.51 | 0.44 | 0.12 | 0.07 | 0.08 | 0.09 | 0.13 | 0.36 | 0.29 | 0.31 | 0.35 | 0.34 |
| | 3 | 84.8 | 72.1 | 69.6 | 70.6 | 54.6 | 1.83 | 1.58 | 1.78 | 1.49 | 1.38 | 2.50 | 2.50 | 2.51 | 2.41 | 2.10 | 0.36 | 0.38 | 0.42 | 0.47 | 0.53 | 0.07 | 0.06 | 0.08 | 0.12 | 0.15 | 0.29 | 0.34 | 0.35 | 0.37 | 0.40 |
| | 4 | 83.8 | 73.4 | 71.6 | 66.1 | 51.8 | 1.47 | 1.81 | 1.51 | 1.57 | 1.27 | 2.16 | 2.25 | 2.20 | 2.07 | 1.97 | 0.50 | 0.43 | 0.45 | 0.39 | 0.55 | 0.08 | 0.08 | 0.09 | 0.11 | 0.16 | 0.33 | 0.35 | 0.36 | 0.37 | 0.32 |
| Lime + Gypsum | 1 | 84.5 | 78.2 | 75.7 | 73.8 | 62.3 | 1.57 | 1.65 | 1.62 | 1.42 | 1.38 | 2.19 | 2.34 | 2.33 | 2.06 | 2.06 | 0.40 | 0.42 | 0.41 | 0.47 | 0.50 | 0.11 | 0.11 | 0.08 | 0.08 | 0.14 | 0.27 | 0.31 | 0.34 | 0.34 | 0.35 |
| | 2 | 84.7 | 78.1 | 76.8 | 74.8 | 64.6 | 1.56 | 1.70 | 1.52 | 1.54 | 1.35 | 2.24 | 2.31 | 2.13 | 2.18 | 1.98 | 0.44 | 0.36 | 0.40 | 0.43 | 0.47 | 0.14 | 0.08 | 0.10 | 0.10 | 0.15 | 0.29 | 0.35 | 0.29 | 0.32 | 0.30 |
| | 3 | 81.7 | 76.7 | 76.2 | 68.8 | 64.0 | 1.55 | 1.54 | 1.60 | 1.52 | 1.32 | 2.23 | 2.21 | 2.28 | 2.16 | 2.01 | 0.47 | 0.44 | 0.43 | 0.44 | 0.53 | 0.10 | 0.11 | 0.08 | 0.08 | 0.16 | 0.35 | 0.32 | 0.33 | 0.37 | 0.37 |
| | 4 | 84.1 | 77.3 | 72.8 | 71.8 | 62.4 | 1.55 | 1.60 | 1.49 | 1.44 | 1.37 | 2.28 | 2.31 | 2.11 | 2.17 | 2.10 | 0.44 | 0.44 | 0.44 | 0.47 | 0.53 | 0.12 | 0.09 | 0.08 | 0.09 | 0.14 | 0.38 | 0.29 | 0.38 | 0.41 | 0.41 |
