# Supplementary material for: Impact of Amendments on the Physical Properties of Soil under Tropical Long-Term No Till Conditions
Source: PLoS One. 2016 Dec 13;11(12):e0167564. doi: 10.1371/journal.pone.0167564 (PMC5154518; doi:10.1371/journal.pone.0167564)
Supplement: S5 Table — (PDF) [file pone.0167564.s005.pdf]

S5. Penetration resistance as affected by surface application of lime and phosphogypsum in different soil layers, in a tropical no-tillage system.

Treatment	Rep	Penetration resistance				
		0-0.05 m	0.05-0.10 m	0.10-0.20 m	0.20-0.40 m	0.40-0.60 m
Control	1	2.64	2.27	2.59	2.19	2.64
	2	2.82	2.44	2.60	2.09	2.59
	3	2.62	2.11	2.67	2.47	2.71
	4	2.21	2.95	2.53	2.01	2.42
Gypsum	1	2.06	2.62	2.50	2.14	2.21
	2	1.97	2.58	2.89	1.83	2.26
	3	2.20	2.90	2.52	1.76	2.11
	4	2.00	2.23	2.63	1.98	1.87
Lime	1	1.51	1.80	2.77	1.98	1.82
	2	1.56	1.99	2.53	2.28	1.62
	3	1.65	2.04	2.92	1.82	1.95
	4	1.51	2.01	2.87	1.82	1.90
Lime + Gypsum	1	1.88	2.16	2.44	2.21	1.74
	2	2.27	1.81	2.18	2.27	1.74
	3	2.02	2.00	2.15	2.14	1.69
	4	1.93	2.01	2.21	1.92	1.80
